# Impact of cataract on health-related quality of life in a longitudinal Japanese chronic obstructive pulmonary cohort

**DOI:** 10.1177/1479972317745735

**Published:** 2017-12-12

**Authors:** Hidehiro Irie, Shotaro Chubachi, Minako Sato, Mamoru Sasaki, Naofumi Kameyama, Takashi Inoue, Yoshitaka Oyamada, Hidetoshi Nakamura, Koichiro Asano, Tomoko Betsuyaku

**Affiliations:** 1 Division of Pulmonary Medicine, Department of Medicine, Keio University School of Medicine, Shinjuku-ku, Tokyo, Japan; 2 Department of Internal Medicine, Sano Kousei General Hospital, Sano, Tochigi, Japan; 3 Department of Respiratory Medicine, National Hospital Organization Tokyo Medical Center, Meguro, Tokyo, Japan; 4 Department of Respiratory Medicine, Saitama Medical University, Iruma-gun, Saitama, Japan; 5 Division of Pulmonary Medicine, Department of Medicine, Tokai University School of Medicine, Isehara-shi, Kanagawa, Japan

**Keywords:** Chronic obstructive pulmonary disease, cataract, quality of life, exacerbation, COPD assessment test

## Abstract

Cigarette smoking increases the risk of developing both cataract and chronic obstructive pulmonary disease (COPD). The prevalence of cataract and the clinical characteristics of COPD patients with cataract were retrospectively investigated in a 2-year observational COPD cohort. We analyzed 395 patients with complete data on ophthalmologic evaluation (319 subjects with COPD and 76 subjects at risk of COPD). There was no difference in the prevalence of cataract between COPD patients and those at risk (47.0% vs. 42.1%, *p* = 0.44). Age ≥ 75 years, low body mass index, and hypertension were independently associated with cataract as a comorbidity in COPD. The incidence of exacerbation within 2 years was significantly higher in COPD patients with cataract than those without cataract (36.6% vs. 18.3%, *p* = 0.0019). COPD patients with cataract exhibited significantly higher COPD assessment test score compared to those without cataract (13.7 ± 8.9 vs. 11.5 ± 7.2, *p* = 0.0240). Overall St George’s Respiratory Questionnaire score and each component were significantly worse in COPD patients with cataract compared to those without cataract. COPD patients with cataract exhibited poor health-related quality of life and frequent exacerbations. The association between cataract and exacerbations of COPD deserves further attention.

## Background

Patients with chronic obstructive pulmonary disease (COPD) frequently suffer from various comorbidities, such as cachexia, lung cancer, cardiovascular disease, osteoporosis, normocytic anemia, diabetes, obstructive sleep apnea, and depression.^1^ In a recent multicenter cohort in Japan, called the Keio COPD Comorbidity Research (K-CCR), we reported the findings of cross-sectional and longitudinal cohort studies that showed associations between some comorbidities and various aspects of COPD.^2–6^ Among a variety of comorbidities, the presence of gastroesophageal reflux disease, depression, arrhythmia, and anxiety was significantly associated with a high COPD assessment test (CAT) score,^2^ implying the importance of a comprehensive approach to COPD comorbidities.

Cataract is the most common cause of reversible loss of useful vision^7^ and is a major public health problem, affecting almost 50% of adults aged >65 years.^8^ In the general population, several factors, such as increasing age, alcohol consumption, poor lifestyle habits, diabetes mellitus, and oral steroid use, are the risk factors for cataract.^9,10^ It should be noted that cigarette smoking is not only a major risk factor for COPD,^11^ but also for cataract.^12,13^ However, cataract has been largely ignored as a comorbidity of COPD.^11,14^ Moreover, the prevalence of cataract and the clinical characteristics of COPD patients with cataract have not been elucidated.

In this study, we focused on cataract, as a comorbidity that pulmonary physicians might routinely pay no attention to COPD. We hypothesized that COPD patients have a higher prevalence of cataract than the general population and that COPD patients with cataract exhibit worse quality of life (QOL). Therefore, we investigated the prevalence of cataract in COPD patients and the differences in clinical characteristics between COPD patients with cataract and those without cataract, as well as the association of cataract with COPD exacerbation within 2-year observation period.

## Patients and methods

### Study population

Our analysis was based on data collected from K-CCR, a prospective observational cohort study that investigated the clinical characteristics of COPD. The study protocol of K-CCR was described elsewhere.^2–4^ A total of 572 subjects were recruited at Keio University Hospital and its affiliated hospitals, including patients who had been diagnosed with COPD and those referred to the investigation of possible COPD. Inclusion criteria were: (i) age ≥ 40 years; (ii) forced expiratory volume in 1 s (FEV_1_)/forced vital capacity (FVC) < 0.7; (iii) presence of emphysematous changes on chest computed tomography (CT); and (iv) chronic respiratory symptoms with significant smoking history (≥30 pack-years). The COPD group fulfilled criteria (i) and (ii), while the non-COPD group met the criteria (i) and either (iii) or (iv) without airflow limitation (FEV_1_/FVC ≥ 0.7). For the specific purpose of this study, the data set of 395 subjects who completed the ophthalmologic evaluation was analyzed. The group at risk of COPD fulfilled the criterion of either the presence of emphysematous changes on chest CT or the presence of chronic respiratory symptoms with significant smoking history (≥30 pack-years). The follow-up period was 2 years. Written informed consent to analyze and present their data was obtained from each patient, and the study (University Hospital Medical Information Network; UMIN000003470) was approved by the ethics committees of Keio University and its affiliated hospitals (20090008).

### Assessment of clinical parameters

Upon enrolment and annually thereafter, full medical and smoking histories, as well as information about current pharmacologic treatment, were obtained based on the reviews of physicians’ medical records and prescription. All patients were assessed by spirometry and chest CT imaging. Spirometry, using an electronic spirometer, was performed in all patients in a stable condition using an electronic spirometer in accordance with the guidelines of the American Thoracic Society.^15^ Predicted values of spirometric measurements were derived from the guidelines issued by the Japanese Respiratory Society for pulmonary function test.^16^ The extent of emphysema was quantified as the ratio of low attenuation area (LAA%) on CT images using custom-made software (AZE Ltd, Tokyo, Japan).^3,6^ Independent investigators in the present study retrospectively judged the number and severity of exacerbations based on the reviews of physicians’ medical records, as we had previously reported.^6^ Mild COPD exacerbation was defined as worsening of symptoms that were self-managed (by measures such as an increase in salbutamol use) and resolved without systemic corticosteroids or antibiotics. Moderate COPD exacerbation was defined as a requirement for treatment with systemic corticosteroids or antibiotics or both. Severe COPD exacerbation was defined as hospitalization, including an emergency admission, for 24 h.

### Assessment of health-related QOL

All patients were clinically stable and had no exacerbations for at least 1 month before study enrollment and the day of annual examinations. The Japanese version of CAT was applied for the assessment of COPD-specific health status,^17,18^ together with the St. George’s Respiratory Questionnaire (SGRQ) in Japanese for the assessment of disease specific instrument with obstructive airways disease.^19–21^ In addition, the Medical Outcomes Study Short-Form 36-Item (SF-36) version 2 was also used to assess the general health status.^22^

### Assessment of cataract and other comorbidities

At enrolment, the diagnosis of cataract was made by ophthalmologic examinations. There was no information about the severity and subtypes of cataract, such as cortical cataract, nuclear cataract, and posterior subcapsular cataract. Other comorbid conditions were diagnosed based on clinical history and physical examination findings, supported by review of available medical records, as we reported previously.^2,3^

### Statistical analysis

Data were presented as mean ± standard deviation (SD) or as median ± interquartile range. Data were compared between two groups using *t*-test, Mann–Whitney *U* test, or *χ* ^2^ test, as appropriate. Univariate and multivariate logistic regression analyses were performed to assess the factors that affect cataract and COPD exacerbations. To compare health-related QOL between the two groups, analysis of covariance (ANCOVA) using age as a covariate was performed. Differences in levels of CAT, SGRQ scores, and in rates of change over time between the two groups were estimated using mixed-effects modeling. Cochran–Armitage trend test was used to identify the age trend of cataract prevalence. Two-sided *p* values of <0.05 were considered significant for all tests. Data were analyzed using JMP 10 software (SAS Institute, Cary, North Carolina, USA).

### Ethical approval

All procedures performed in studies involving human participants were in accordance with the ethical standards of the institution and/or research committee and with the 1964 Helsinki declaration and its later amendments or comparable ethical standards (clinical trial registered with UMIN (UMIN000003470)).

### Informed consent

Written informed consent to analyses and present their data was obtained from each patient

## Results

### Patient characteristics

The study population comprised of 319 COPD patients and 76 individuals who were at risk of COPD. The baseline characteristics of the patients are shown in Table 1. Compared with the group at risk of COPD, patients with COPD were older (*p* = 0.0014) and had higher pack-years of smoking (*p* = 0.0386). The average age of COPD patients (72 years) was older than that of a Western COPD cohort.^23^ At baseline, 22.9%, 46.1%, 24.1%, and 6.9% were diagnosed as COPD grade, the Global Initiative for Chronic Obstructive Lung Disease (GOLD) I, II, III, and IV, respectively.

**Table 1.** Characteristics of the study population.^a^

	At risk of COPD	COPD	*p*-Value
*N*	76	319	
Gender, female, *n* (%)	6 (7.9)	35 (11.0)	0.429
Age (years)	67.9 ± 11.4	72.4 ± 7.8	0.0014
Smoking index (pack-years)	46.5 ± 29.0	54.6 ± 29.8	0.0386
Current smokers, *n* (%)	22.6 ± 4.4	22.7 ± 3.4	0.864
BMI (kg/m^2^)	22.6 ± 4.4	22.7 ± 3.4	0.864
FEV_1_/FVC (%)	76.8 ± 5.9	51.7 ± 12.3	<0.0001
%FEV_1_ (%)	88.6 ± 17.5	62.7 ± 21.7	<0.0001
GOLD grade I/II/III/IV, *n* (%)		73/147/77/22 (22.9/46.1/24.1/6.9)	

BMI: body mass index; FEV_1_: forced expiratory volume in 1 s; FVC: forced vital capacity; %FEV_1_: ratio of predicted FEV_1_; GOLD: Global Initiative for Chronic Obstructive Lung Disease; COPD: chronic obstructive pulmonary disease; SD: standard deviation.

^a^Data are shown as numbers (%) and mean ± SD.

### Prevalence of cataract

**Figure 1.**
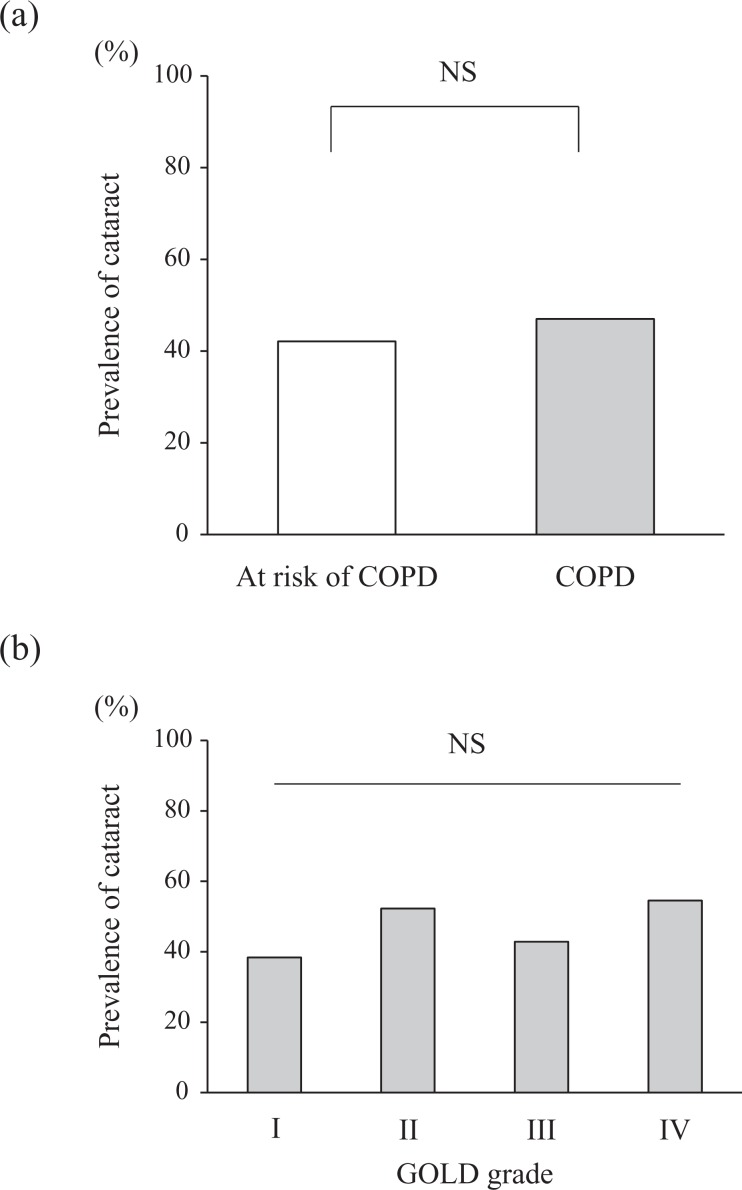
Prevalence of cataract according to diagnosis or risk of COPD and GOLD grade. COPD: chronic obstructive pulmonary disease; GOLD: Global Initiative for Chronic Obstructive Lung Disease; NS: not significant.

The prevalence of cataract is shown in Figure 1. The prevalence of cataract was the same between COPD patients and COPD at risk subjects (47.0% vs. 42.1%, respectively, *p* = 0.44; Figure 1(a)) and among the different GOLD grades of COPD (Figure 1(b)).

### Characteristics of COPD patients with cataract

**Figure 2.**
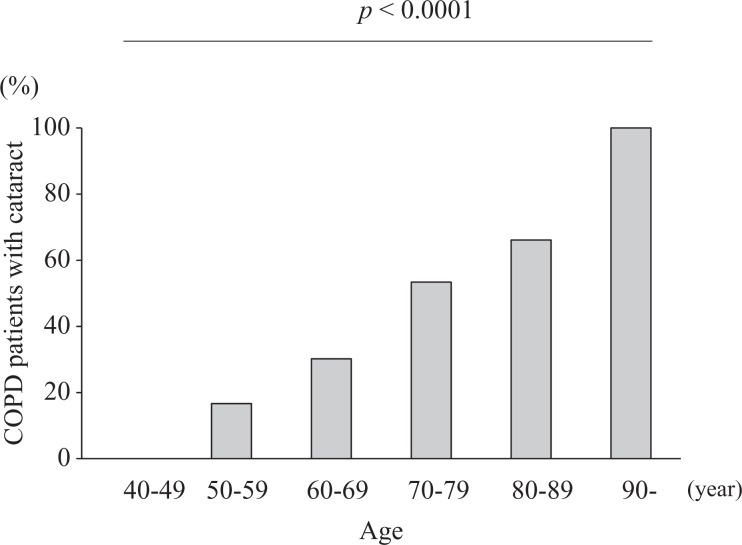
Proportion of COPD patients with cataract according to age group, *Z* = 5.406 and *p* < 0.0001. COPD: chronic obstructive pulmonary disease.

The characteristics of COPD patients stratified by the presence of cataract are shown in Table 2. Patients with cataract were significantly older than those without cataract (75.0 ± 6.8 vs. 70.1 ± 8.0, *p* < 0.0001). There were more women than men who had cataract (14.7% vs. 7.7%, *p* = 0.0467). The Cochran–Armitage trend test showed that cataract prevalence increased significantly with age (*Z* = 5.406 and *p* < 0.0001; Figure 2). The mean body mass index (BMI) was significantly lower in patients with cataract than in those without cataract (22.3 ± 3.3 vs. 23.2 ± 3.4, *p* = 0.017). Among the lifestyle-related diseases, hypertension had significantly higher prevalence in COPD patients with cataract than those without cataract (47.3% vs. 32.9%, *p* = 0.0096). There were no differences in pack-years, the ratio of the predicted FEV_1_(%FEV_1_), LAA%, and other comorbidities between the two groups. In addition, there was no differences in the ratio of prescribed inhaled corticosteroids (ICSs) and dose of ICS between the two groups.

**Table 2.** Clinical characteristics of COPD patients according to the presence of cataract.^a^

	Patients without cataract	Patients with cataract	*p*-Value
*N*	169	150	
Gender, female, *n* (%)	13 (7.7)	22 (14.7)	0.0467
Age (years)	70.14 ± 8.0	75.0 ± 6.8	<0.0001
Smoking index (pack-years)	55.3 ± 29.8	53.8 ± 29.8	0.666
Current smokers, *n* (%)	19 (11.7)	14 (9.6)	0.557
BMI (kg/m^2^)	23.2 ± 3.4	22.3 ± 3.3	0.017
%FEV_1_ (%)	64.5 ± 22.0	60.6 ± 21.2	0.111
GOLD grade I/II/III/IV, *n* (%)	45/70/44/10 (26.6/41.4/26.0/5.9)	28/77/33/12 (18.7/51.3/22.0/8.0)	0.177
LAA% (%)	10.6 (5.4–22.7)	13.0 (5.2–22.5)	0.817
Comorbidities			
Diabetes mellitus, *n* (%)	29 (17.7)	23 (15.5)	0.612
Hyperuricemia, *n* (%)	13 (7.9)	18 (12.2)	0.212
Hypertension, *n* (%)	54 (32.9)	70 (47.3)	0.0096
Dyslipidemia, *n* (%)	35 (21.3)	26 (17.6)	0.401
Coronary artery disease, *n* (%)	22 (13.4)	13 (8.8)	0.1956
Steroid use			
ICS, *n* (%)	49 (29.5)	50 (33.3)	0.172
Dose of ICS,^b^ µg/day, median	500	500	0.125
OCS, *n* (%)	5 (3.0)	5 (3.4)	0.854

BMI: body mass index; COPD: chronic obstructive pulmonary disease; %FEV_1_: ratio of predicted FEV_1_; GOLD: Global Initiative for Chronic Obstructive Lung Disease; LAA%: ratio of low attenuation area; ICS: inhaled corticosteroid; OCS, oral corticosteroids; SD: standard deviation.

^a^Data are shown as mean ± SD and median (interquartile range).

^b^Dose of ICS is shown as fluticasone propionate equivalent.

### Association of cataract and health status in COPD patients

**Figure 3.**
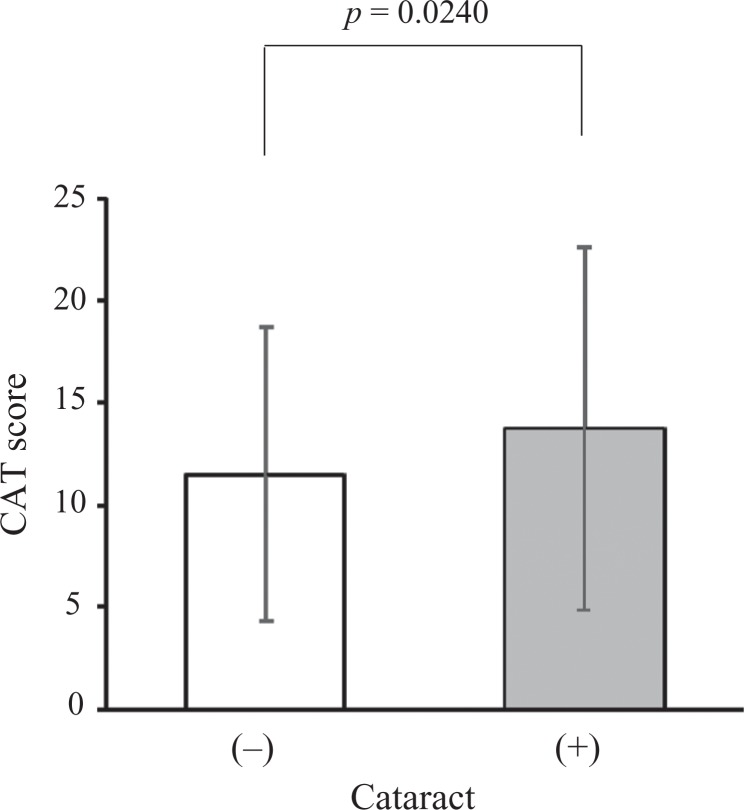
Comparison of baseline mean CAT score in COPD patients according to the presence or absence of cataract. COPD: chronic obstructive pulmonary disease; CAT: COPD assessment test.

**Figure 4.**
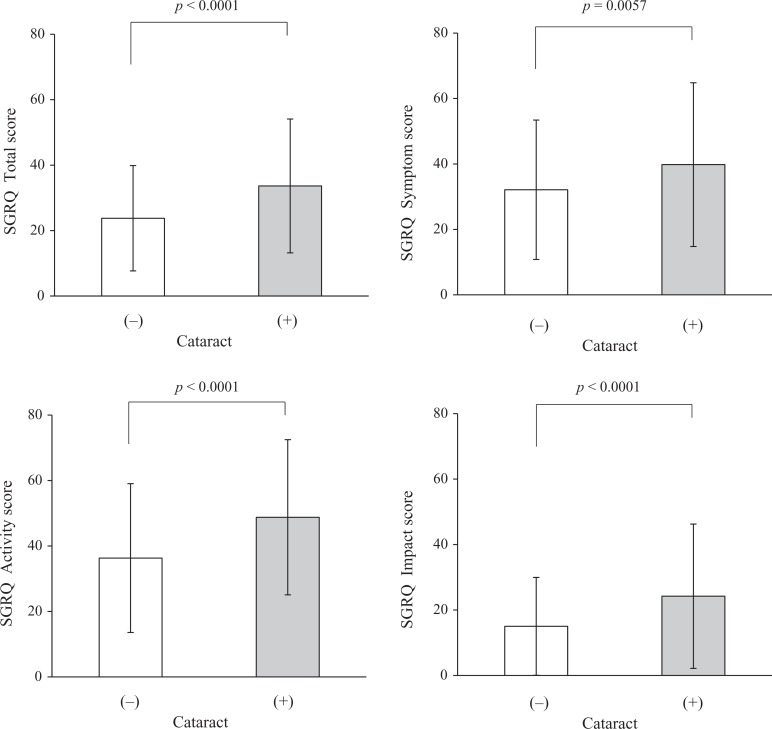
Comparison of baseline mean overall and individual SGRQ scores in COPD according to the presence or absence of cataract. COPD: chronic obstructive pulmonary disease; SGRQ: St George’s Respiratory Questionnaire.

At baseline, COPD patients with cataract exhibited significantly higher total CAT score compared with those without cataract (13.7 ± 8.9 vs. 11.5 ± 7.2, *p* = 0.0240; Figure 3). In addition, overall SGRQ score and each of its components were significantly worse in COPD patients with cataract compared to those without cataract (Figure 4). In a similar way, SF-36 physical functioning, physical role functioning, general health perception, social role functioning, and emotional role functioning scores were worse in COPD patients with cataract compared to those without cataract (Online Supplemental Figure S1). We assessed the effect of having cataract on CAT and total SGRQ scores after the adjustment of age. ANCOVA showed significant age-adjusted differences in CAT and total SGRQ scores between two groups (CAT: *p* = 0.047 and SGRQ: *p* = 0.0003).

**Figure 5.**
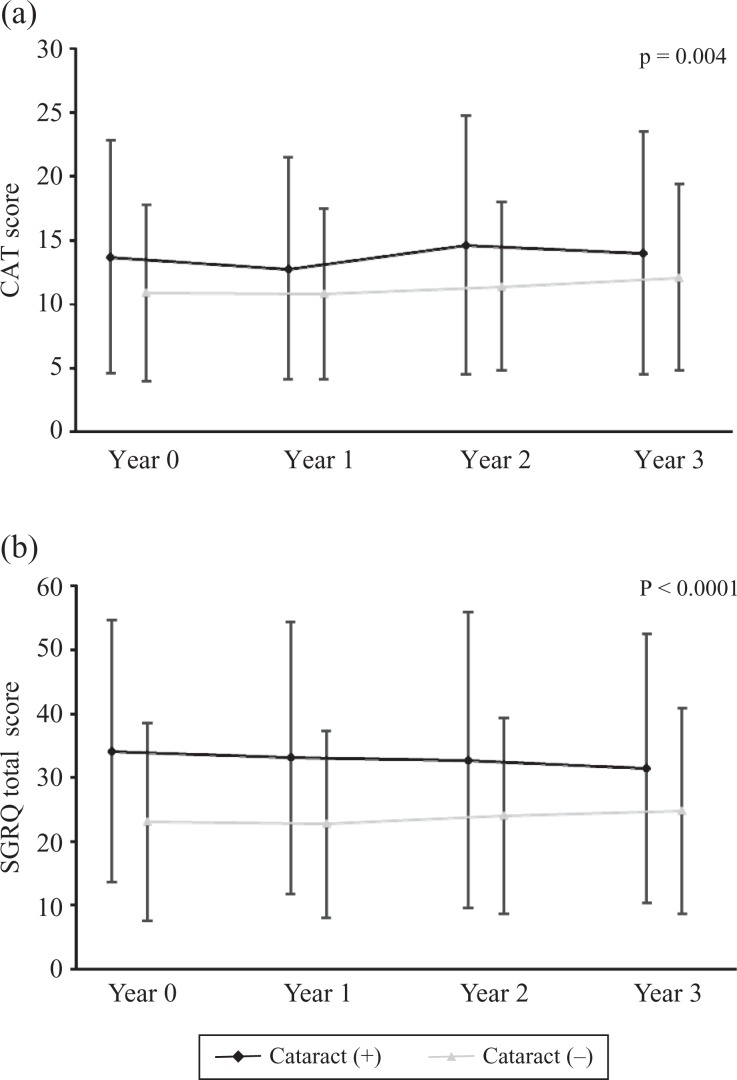
Follow-up analysis of the difference in (a) CAT scores and (b) total SGRQ scores within 3 years according to the presence or absence of cataract. CAT: chronic obstructive pulmonary disease assessment test; SGRQ: St George’s Respiratory Questionnaire.

Follow-up analysis within 3 years indicated that the difference in total CAT scores and total SGRQ scores between the two groups was also significant (CAT: *p* = 0.004 and SGRQ: *p* < 0.0001; Figure 5). However, there was no significant difference in the rate of change in these scores between the two groups over 3 years (CAT: *p* = 0.744 and SGRQ: 0.561).

### Predictors of cataract development in COPD patients

Based on the univariate logistic regression analysis, COPD patients with cataract were more likely to be ≥75 years of age, female, with low BMI, and having hypertension (Online Supplemental Table S1). Multivariate logistic regression analyses were performed based on a model of risk factors that reached the statistical significance in the univariate analyses (Table 3). Age ≥ 75 years, low BMI, and hypertension were independently associated with the presence of cataract in COPD patients.

**Table 3.** Predictors of cataract by multivariate logistic regression analysis.

	Odds ratio (95% CI)	*p*-Value
Gender, female	1.78 (0.83–3.93)	0.1382
Age ≥ 75 years	2.32 (1.45–3.76)	0.0005
BMI	0.16 (0.04–0.62)	0.0081
Hypertension	1.83 (1.12–3.00)	0.0162

BMI: body mass index.

### Association of cataract and COPD exacerbations

**Figure 6.**
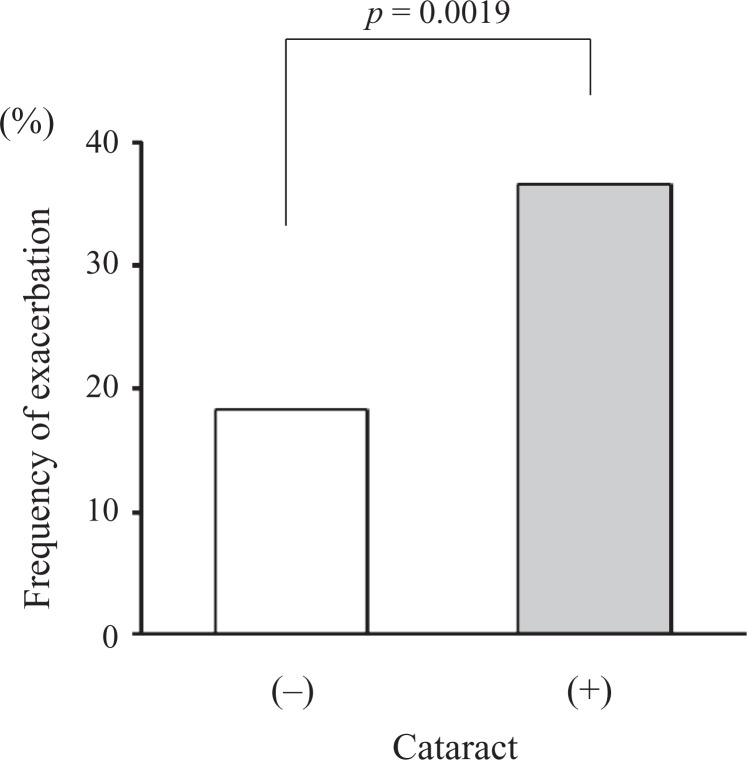
Frequency of COPD exacerbations within 2 years according to the presence or absence of cataract. COPD: chronic obstructive pulmonary disease.

The incidence of moderate and severe exacerbations over 2 years was significantly higher in COPD patients with cataract than those without cataract (36.6% vs. 18.3%, *p* = 0.0019; Figure 6). After adjusting for age ≥ 75 years, gender, and %FEV_1_ < 50%, having cataract increased the number of COPD exacerbations over 2 years (odds ratio = 2.99, 95% confidence interval: 1.57–5.90, and *p* = 0.0008). In the COPD patients, who experienced moderate or severe exacerbations over 2 years, there was no significant difference in the oral steroid use between the patients with cataract and those without cataract (1/23 vs. 5/34, *p* = 0.21).

## Discussion

To the best of our knowledge, this was the first and largest scale study that investigated the prevalence of cataract in COPD and the clinical characteristics of COPD patients with cataract. There were some previous studies, which showed that cigarette smoking increased the risk of cataract.^12,13^ However, the relationship between cataract and COPD had been largely overlooked. In addition, the possible risk factors of cataract development in COPD patients and the influence of cataract on health-related QOL of COPD patients had not been investigated.

Visually significant cataract is present in approximately 42.8% of Americans aged 70–79 years.^24^ This prevalence was completely in line with the findings of a Japanese age-related cataract epidemiologic study on a general population.^25^ In this study, ophthalmologic examinations objectively revealed that cataract prevalence was 53.38% in COPD patients aged 70 to 79 years; this prevalence was equivalent to that of subjects at risk of COPD. In the general population, older age, cigarette smoking, female, steroid use, and diabetes mellitus are well-established risk factors for cataract.^7,9,10,12,13^ In the present study, older age, low BMI, and hypertension were the independent risk factors for cataract in COPD patients; however, smoking, gender, steroid use, and diabetes mellitus were not associated with cataract in this present cohort of COPD patients. In addition, COPD severity according to pulmonary function or degree of emphysema was not related to the presence of cataract, suggesting that cataract was not a comorbidity that developed along with the progression of COPD.

Our study provided two novel observations of potential relevance. First, cataract in COPD patients was associated with worse QOL measures, not only SF-36, but also CAT and SGRQ. In the general population, visual impairment caused by eye diseases, such as cataract, had worse impact on SF-36.^26^ Our findings suggested that the treatment of cataract may improve visual acuity, aspects of daily living, and overall QOL. The definitive treatment for cataract is the surgical removal and replacement of the opacified eye lens to restore the transparency of the visual axis. Modern surgical techniques have been safely performed with few major complications.^27^ Second, exacerbations were more frequent in COPD patients with cataract than those without cataract during the 2 years of follow-up. Frequent exacerbation is a major driver of worsening health status^6,28^ in COPD and is an important cause of hospital admission^29^ and death.^30^ The pathophysiology of COPD exacerbation is complex.^31–33^ In the present study, having cataract turned out to be an independent risk factor for future exacerbations, even after adjusting the known risk factors of exacerbations.^33,34^ Among the COPD patients, there was no significant difference in inhaled steroid dose between patients with cataract and those without. In the COPD patients, who experienced moderate or severe exacerbation, there was also no difference in the ratio of oral steroid use between the group of patients with cataract and those without. At least in this population, steroid use was not associated with the presence of cataract. The specific cause and effect relationship between cataract and exacerbations is unclear. However, the development of cataract and COPD exacerbation may include a common pathogenesis, such as robust oxidative stress.^35,36^ In addition, the poor health-related QOL of COPD patients with cataract may be indirectly related to frequent exacerbations.

Some studies showed that the use of higher doses and longer duration of ICS is associated with the prevalence of cataracts in COPD patients.^1,2^ However, there were no differences in ICS use between COPD patients with cataracts and those without in this study. The reason of the discrepancy might be lower dosage of daily ICS and relatively smaller number of subjects in our study compared to the previous studies.^37,38^

One of the major strengths of this study was the comprehensive assessment of comorbid factors in the K-CCR cohort study and the longitudinal follow-up.^6,39^ In this study, we analyzed only data from patients who had completed the ophthalmologic examinations at enrollment. The development of age-related cataract is sometimes asymptomatic, but progresses by age. Lens opacity can only be confirmed by a fundus examination using the direct ophthalmoscope. In some previous studies, the presence of cataract was defined as any database code for cataract without ophthalmologic examinations.^40,41^ Therefore, it might be possible that patients who had incomplete cataract with slightly opaque lens and clear cortex were not considered as having cataract in those previous studies and might have affected the results. Nevertheless, increasing the awareness of both patients and pulmonary physicians about cataract as a comorbid condition of COPD should be emphasized.

There were several limitations of the present study. First, this study did not obtain information on surgery, types, grades of cataract,^7,42^ and proportion of patients with visual impairment. Differentiation among the several sites of cataract formation (i.e. central lens location and granular opacification) is important, because posterior subcapsular cataract is more likely to result in visual impairment at an earlier stage and would be more likely to impair function and require early surgical intervention.^7,43,44^ Second, Japan has become a super-aged society before other countries did^45^; the average age of patients who participated in our study was higher than that of other COPD clinical studies conducted in Western countries. Increasing age has been constantly associated with nuclear and cortical opacities; therefore, when clinicians apply the results of this study to a general COPD patient population, aging factor should be kept in mind.

## Conclusions

COPD patients with cataract exhibited worse health-related QOL, and cataract was shown to be related to frequent COPD exacerbations. The pathophysiologic association between cataract and COPD exacerbation deserves further investigation.

## Supplemental material

supplemental_fig1 - Impact of cataract on health-related quality of life in a longitudinal Japanese chronic obstructive pulmonary cohort Click here for additional data file. supplemental_fig1 for Impact of cataract on health-related quality of life in a longitudinal Japanese chronic obstructive pulmonary cohort by Hidehiro Irie, Shotaro Chubachi, Minako Sato, Mamoru Sasaki, Naofumi Kameyama, Takashi Inoue, Yoshitaka Oyamada, Hidetoshi Nakamura, Koichiro Asano, Tomoko Betsuyaku, and On behalf of the Keio COPD Comorbidity Research (K-CCR) Group in Chronic Respiratory Disease
