# Supplementary figures and images for: Impact of cataract on health-related quality of life in a longitudinal Japanese chronic obstructive pulmonary cohort
Source: Chron Respir Dis. 2017 Dec 12;15(4):329–38. doi: 10.1177/1479972317745735 (PMC6234576; doi:10.1177/1479972317745735)

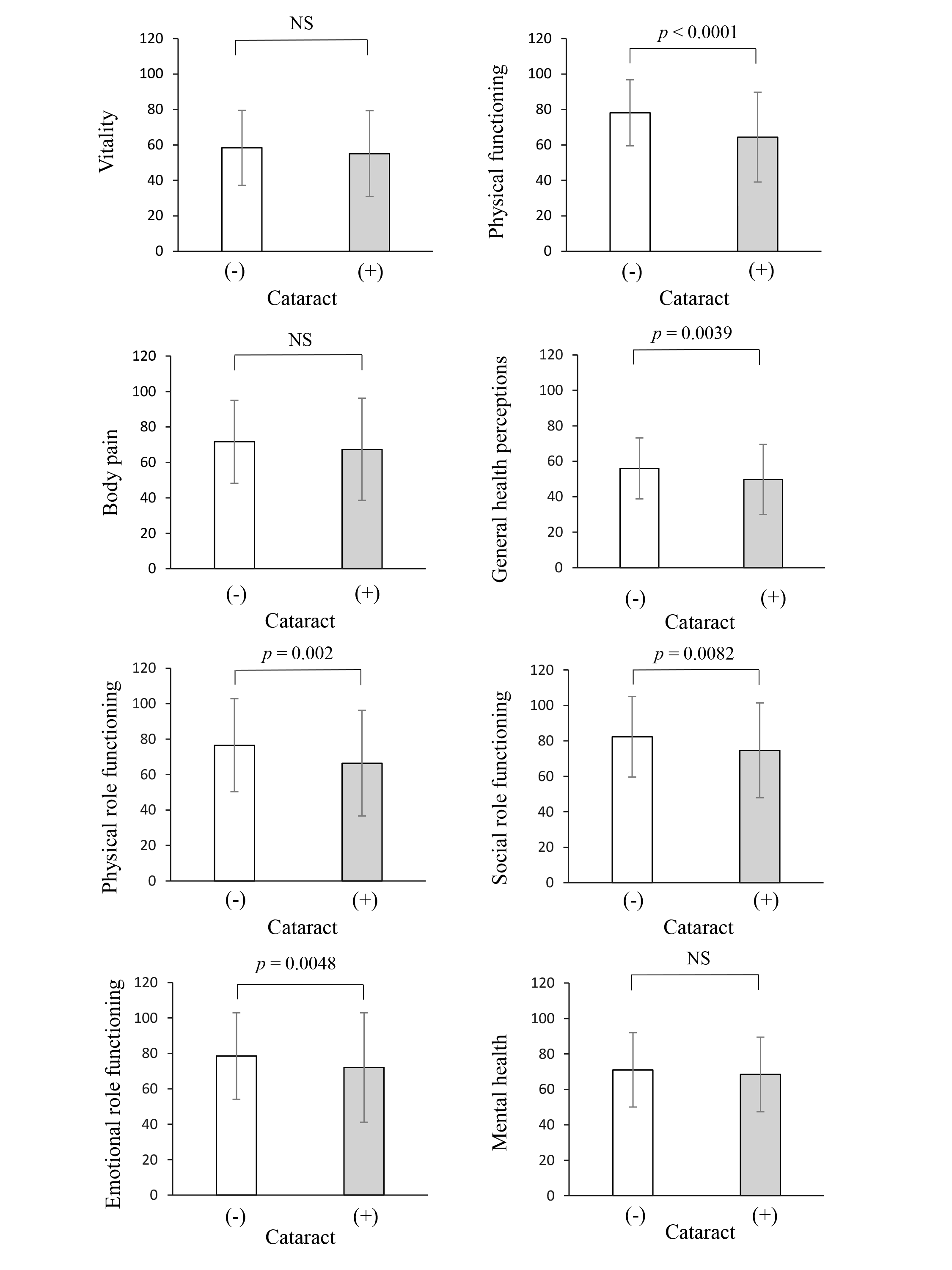

Supplement: supplemental_fig1 - Impact of cataract on health-related quality of life in a longitudinal Japanese chronic obstructive pulmonary cohort [file supplemental_fig1.TIF]
